# Application of vibration to the soles reduces minimum toe clearance variability during walking

**DOI:** 10.1371/journal.pone.0261732

**Published:** 2022-01-04

**Authors:** Prabhat Pathak, Jeongin Moon, Se-gon Roh, Changhyun Roh, Youngbo Shim, Jooeun Ahn

**Affiliations:** 1 Department of Physical Education, Seoul National University, Seoul, Republic of Korea; 2 Robot Center in Samsung Seoul R&D Campus, Samsung Electronics Co., Ltd., Seoul, Republic of Korea; 3 WI Robotics, Suwon, Gyeonggi, Republic of Korea; 4 T-Robotics, Osan, Gyeonggi, Republic of Korea; 5 Institute of Sport Science, Seoul National University, Seoul, Republic of Korea; University of Pittsburgh, UNITED STATES

## Abstract

Minimum toe clearance (MTC) is an important indicator of the risk of tripping. Aging and neuromuscular diseases often decrease MTC height and increase its variability, leading to a higher risk of tripping. Previous studies have developed visual feedback-based gait training systems to modify MTC. However, these systems are bulky and expensive, and the effects of the training continue only for a short time. We paid attention to the efficacy of vibration in decreasing the variability of gait parameters, and hypothesized that proper vibration applied to soles can reduce the MTC variability. Using shoes embedded with active vibrating insoles, we assessed the efficacy of both sub- and supra-threshold vibration in affecting MTC distribution. Experiment results with 17 young and healthy adults showed that vibration applied throughout the walking task with constant intensity of 130% of sensory threshold significantly decreased MTC variability, whereas sub-threshold vibration yielded no significant effect. These results demonstrate that a properly designed tactile sensory input which is controlled and delivered by a simple wearable device, the active insole, can reduce the MTC variability during walking.

## Introduction

In human walking, reliable control of the foot position is essential for avoiding any accidental ground contact that might result in falls [1, 2]. The kinematics of the swing foot is especially important in that the foot travels with considerable momentum while the whole body is supported by a single foot during the swing phase. In particular, for a stereotyped human gait, the foot is most likely to hit the ground when the foot is at the lowest distance from the ground during swing phase. The distance between the ground and the toe at this position is termed minimum toe clearance (MTC), which is accepted as a critical biomechanical indicator of tripping [3–5]. Aging and neurological diseases are primarily known to have detrimental effects on MTC, lowering the average value and increasing the variability, which leads to a higher risk of tripping [6–8]. Methods for mitigating these changes in MTC are necessary.

Several studies developed training systems with real-time visual feedback to modify MTC during walking. Tirosh et al. trained healthy young adults to increase their MTC by visualizing the toe trajectory and instructing them to follow a template of MTC values for 10 minutes [9]. The retention period of the modified MTC was 10 minutes. Begg et al., using a similar system, trained participants with stroke and older adults for 5 minutes to reduce MTC variability; the effect was retained for 5 minutes after the training [10]. Although these systems showed efficacy, they require bulky and expensive equipment, including a large screen and a treadmill. The training also requires a long-term routine, and the beneficial effect lasts only for a limited period of time.

We propose that applying vibration to soles using a compact wearable device can resolve some important drawbacks of the aforementioned systems. Walking requires afferent feedback from numerous sensory organs to make adjustments to changes in the environment and regulate balance [11–13]. In particular, the mechanoreceptors (i.e., Ruffini, Pacinian, and Meissner’s corpuscles, and Merkel disks) located at the plantar surface of the foot are a rich source of somatosensation and proprioception during locomotion [13, 14]. This important end-effector is also the closest point of interaction with the external environment during steady-state walking, which provides crucial information regarding the walking surface for the central nervous system [15, 16]. Previous studies have shown that reduction in afferent feedback from the soles of the feet alters rhythmic gait pattern and lower limb muscles activity, linked to degradation of balance [16–18]. On the other hand, numerous studies have shown that non-noxious stimulation to innervate or directly stimulate mechanoreceptors at the soles can modulate lower limb muscle reflexes [19–21], which can be utilized to fine tune the motor control system during walking. This stimulation induced fine-tuned reflex actions are shown to adjust the lower limb trajectories and muscle activations that contribute to avoiding obstacles during the swing phase [15, 22, 23]. Hence, sensory input to these important end-effector receptors can provide additional cutaneous feedback to beneficently affect locomotion.

Adding noisy but proper mechanical vibration over a large frequency range can amplify the stimuli, enabling the sensory system to detect weak signals; the vibration, which is applied to the plantar surface of the feet with the amplitude slightly below or above the sensory threshold, enhances human motor performance effectively [24–26]. Previous studies have shown that vibration stimulation on the soles can improve standing balance of healthy young adults, the elderly, individuals with stroke, and patients with diabetic neuropathy [27–29]. The effect of vibration on human gait has also been investigated. Multiple studies have shown that the application of sub-threshold vibration on the soles decreases the variability of stride time, length, and width [30–32]. Recently, Yamashita et al. applied supra-threshold vibration to the plantar surface of the foot to reduce the variability of foot trajectory during walking [33]. However, the effects of both sub-threshold and supra-threshold vibration specifically on MTC have not been systematically addressed.

Here, we hypothesize that proper mechanical vibration applied to the soles can affect MTC distribution. We particularly considered using insoles embedded with vibrating actuators as a practical method for providing the proper intervention. In our previous study, we equipped shoes with insoles containing piezoelectric actuators and showed that sub-threshold vibration delivered by these shoes could mitigate fatigue-induced declines in balance ability [34]. Unlike the bulky and wired systems that provided necessary interventions in other previous studies, these shoes can provide the vibration even outside the laboratory environment; the power is supplied by a rechargeable battery built in the shoes, and the vibration is controlled wirelessly using a smartphone application and Bluetooth technology. This compactness makes the shoes readily available for daily use. In this study, we aim to evaluate the efficacy of such a compact and practical technology in reducing the MTC variability during walking. Furthermore, to determine the more effective level of stimulus, we applied both sub and supra-threshold vibrations and investigated the effect of each on MTC variability. Other vibration-induced changes in the variability of three-dimensional lower limb joint angles and its association with MTC variability were additionally investigated.

## Materials and methods

### Participants

Seventeen healthy young adults (9 Males and 8 Females; age: 28.29±5.17 years; height: 168.65±6.74 cm; weight: 65.12±13.32 kg) participated in the study. The number of participants in our study is higher than the estimated sample size required to elicit 95% statistical power. We initially set the effect size as 0.51 based on a previous study [35], and selected p-value for statistical significance and expected power as 0.05 and 0.95, respectively. Under this condition, G-power software [36] estimated the sample size as 12. The participants had no known neuromuscular, orthopedic, and cardiovascular disorders. We determined the dominant foot of each participant as the foot that the participant prefers to use when kicking a ball. The institutional review board (IRB) of Seoul National University approved all aspects of the study, which conformed to the principles and guidelines described in the Declaration of Helsinki (IRB No. 2004/001-016). Participants provided informed, written consent before participation.

### Equipment

**Fig 1:**
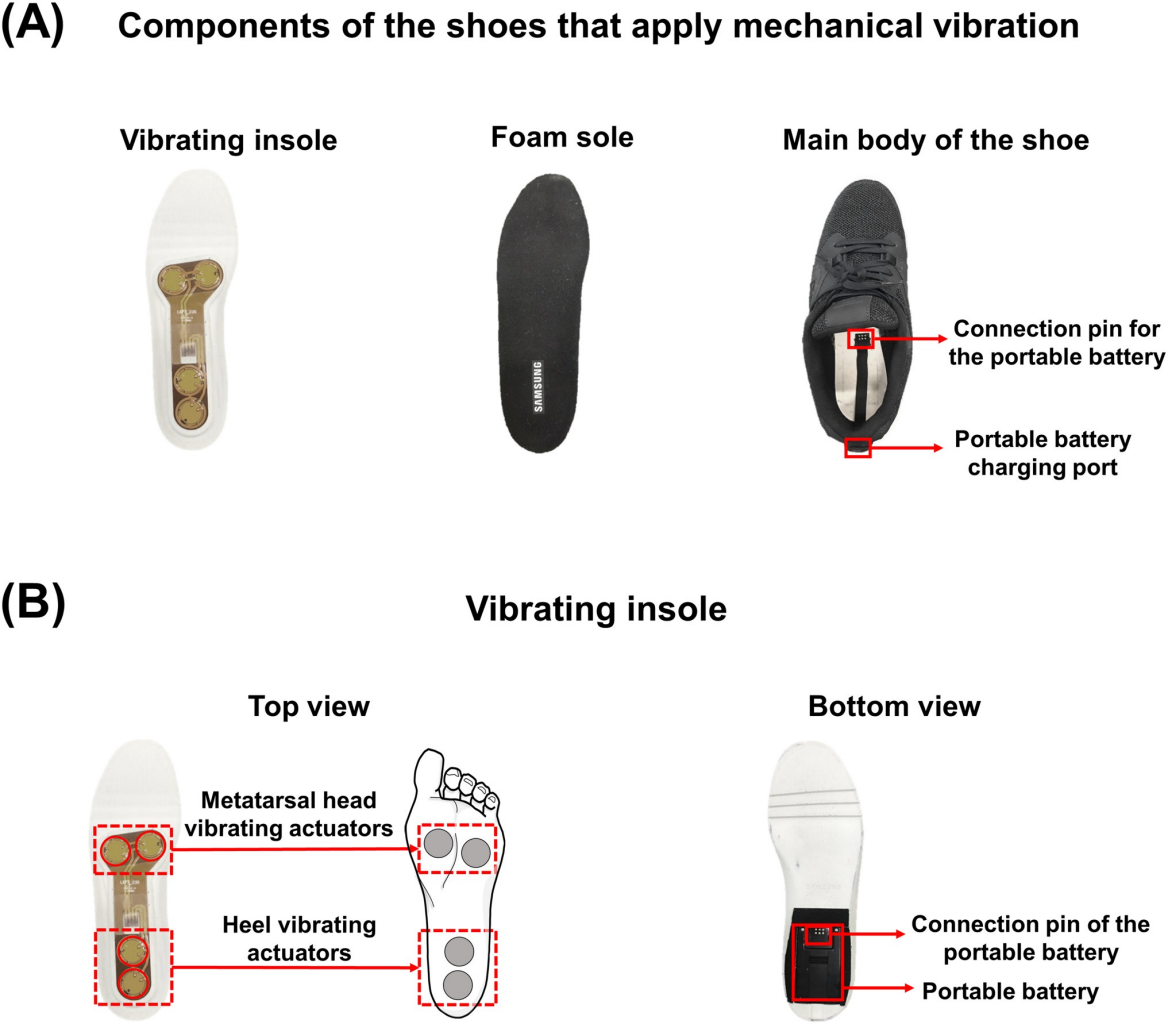
Overview of the components of the shoes and vibrating insole that apply mechanical vibration. (A) The components of the shoes that apply the mechanical vibration to the soles of the feet. The main components are the vibrating insoles that contains the actuators that deliver mechanical vibration, a foam sole placed on top of the insoles to act as a shock absorber, and main body of the shoe that contains the connection pin and charging port to recharge battery. (B) The top and bottom view of the vibrating insoles. The top view shows the position of pair of actuators used to deliver the vibration to the metatarsal head (front actuators) and heel (rear actuators). The amplitude of the vibration is controlled independently for the two pairs of actuators, which allows users to apply the vibration to different areas of the foot with different amplitudes. The bottom view shows the position of the portable battery and the connection pin for the charging port of the shoes.

The mechanical vibration was delivered to the metatarsal head and heel sections of the feet using a pair of insoles embedded with four piezoelectric actuators and a rechargeable battery (Fig 1). Each insole is placed inside a custom-built shoe, which contains a charging port for the battery. The amplitude of the vibration applied to each of four different locations (the metatarsal head or heel of the left or right foot) was independently controlled wirelessly via custom-built smartphone software. Further detailed specifications of the piezoelectric actuators, shoes, and smartphone software are available in [34].

The walking task was performed on a pressure pad embedded treadmill (Model Gait analysis FDM-TDSL-3i, Zebris Inc^®^, Germany; belt length: 150 cm; belt width: 50 cm; maximum incline: 15%; maximum speed: 24 km/hr). Ten infra-red cameras (Optitrack Prime^X^ 13, Natural Point, Inc., Oregon, USA) were used to record the position of the reflective markers placed on the joints in medial-lateral (X), anterior-posterior (Y), and proximal-distal (Z) directions at a sampling frequency of 100 Hz.

### Experimental procedure

We provided the participants with athletic attire to wear during the experiment. To estimate the preferred walking speed (PWS) of the participants, we initially asked them to walk at a speed of 2.5 km/hr, and increased the speed by 0.1 km/hr per 10 seconds. We requested the participants to report once they detected the speed that best characterized their everyday walking speed. After the participant reported the speed for the first time, we instantaneously increased the speed by 1.0 km/hr and decreased it by 0.1 km/hr per 10 seconds until the participants detected the speed that best characterized their everyday walking speed. Considering that variations in PWS may affect the experimental results, we repeated this measuring process three times, and took the average speed as the PWS.

For each participant, we determined the sensory threshold of the mechanical vibration for each section of the foot separately in a sitting position before the walking task. The method for determining the sensory threshold is described in detail in our previous study [34]. To recap here briefly, we applied the vibration in each section of the foot in incremental order, and the participants were asked to report once they sensed the vibration. This procedure was repeated three times, and the average value was used as the sensory threshold. Because each of the metatarsal head and heel of both feet has different threshold, we estimated the threshold of each area separately in a random order. The sensory threshold was determined in a sitting position, although the threshold is higher in standing position [37]. This was because standing requires continuous shift in weight and joint movement to maintain balance, which might lead to unreliable measurement of the sensory threshold. After determining the sensory threshold, we placed 20 reflective markers on anatomical landmarks at the dominant and non-dominant legs: the heel, first metatarsal, fifth metatarsal, medial and lateral malleolus, medial and lateral epicondyle, greater trochanter, and anterior and posterior superior iliac spine. The positions of the markers were used to calculate the MTC and joint angles.

The participants performed walking tasks at their PWS under three conditions: no vibration (No), sub-threshold vibration (Sub), and supra-threshold vibration (Supra). Consulting previous studies which showed motor performance augmentation under the vibration with the amplitude of 90% and 130% of the sensory threshold [38], we respectively selected the amplitude of vibration for Sub and Supra condition as such. Before starting the walking trial, we recorded the data of standing for three seconds to calibrate the marker positions. We randomized the order of the experimental conditions, and provided 5 minutes of rest between trials. Considering the typical time required for adaptation to a treadmill system [39], we asked the participants to walk for 10 minutes in the first trial, and 7 minutes each in the second and third trials, and analyzed the data acquired for the last 5 minutes for all the trials. Under Sub and Supra conditions, the vibration was turned on throughout the last 5 minutes of walking trials. Considering that the sensory threshold while standing or walking is higher than that while sitting, we asked participants during the rest period whether they had perceived the vibration after the end of each trial. All the participants reported that they felt the vibration under the Supra condition, whereas none of them felt it under the Sub condition.

### Data processing

The raw coordinates of the reflective markers were filtered using a zero-lag low pass Butterworth filter with a cut-off frequency of 10 Hz. After that, Visual 3D (Visual3D v6^™^, C-Motion, Inc., Maryland, USA) was used to build a seven-segment model (the dominant and non-dominant foot, shank and thigh, and the single pelvis) to calculate joint kinematics.

#### Minimum toe clearance (MTC) height and variability

**Fig 2:**
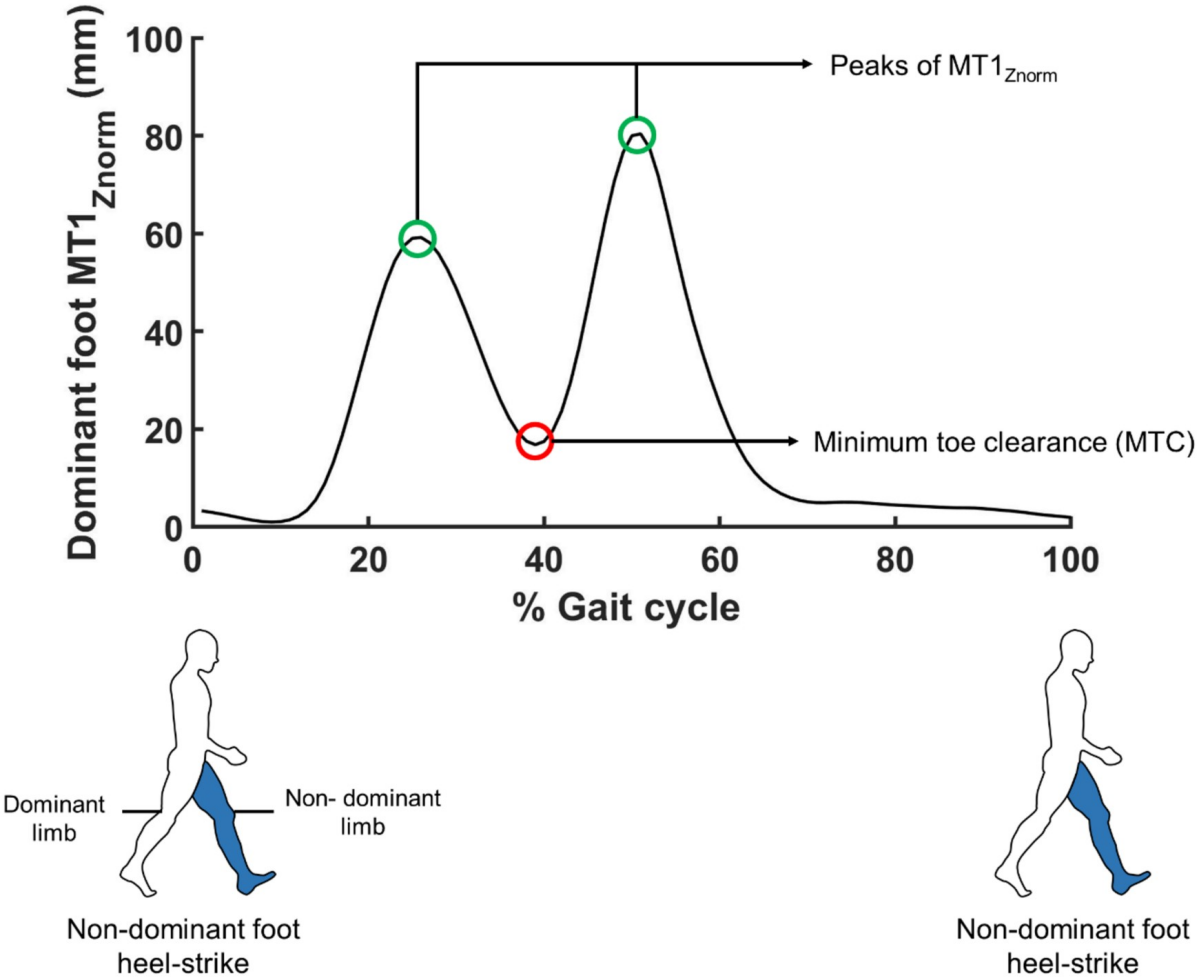
Illustration of the process of detecting MTC. MTC is obtained from the trajectory of the reflective marker attached to the first metatarsal in the vertical direction (MT1_Z_). The average MT1_Z_ during the three-second standing is set as the baseline position of the toe. This average value is subtracted from the entire original MT1_Z_ profile and the resulting profile is labeled as MT1_Znorm_. Each gait cycle of one leg is defined as the period from a heel-strike (HS) to the subsequent HS of the other leg. (The figure shows the example of one gait cycle defined to calculate MTC of the dominant foot.) The MT1_Znorm_ during one gait cycle exhibits two peaks (green circles). MTC is defined as the lowest MT1_Znorm_ between these two peaks (red circle).

The process of detecting MTC is illustrated in Fig 2. The trajectory of the marker attached at the first metatarsal in Z-direction (MT1_Z_) was used to evaluate MTC. We first calculated the ground position of the toe by averaging the value of MT1_Z_ during the three-second standing trial. The MT1_Z_ during the entire 5 minutes data acquisition was then subtracted from the ground position of the toe, and we labeled this quantity as MT1_Znorm_. We defined the gait cycle of the dominant foot as the period between the heel strike (HS) and the subsequent HS of the non-dominant foot. The gait cycle of the non-dominant foot was defined vice-versa. Consulting a previous study [40], the moment of heel strike was defined as the time point when the distance between the position of the heel marker and pelvis center of mass reaches the local maximum. The 17 participants walked between 244 and 278 strides during the 5 minutes data acquisition period. The MT1_Znorm_ trajectory of each foot during each gait cycle exhibited two peaks. Each local minimum of MT1_Znorm_ between the peaks is defined as MTC height during each gait cycle. We then calculated the average and standard deviation of the MTC heights during the 5 minutes.

#### Three-dimensional lower limb joint angles

We calculated the three-dimensional angles of the ankle, knee, and hip, separately for the dominant and non-dominant limbs, using the Visual 3D software. The coordinate system of the joint angle follows the default Cardan sequence of the software: flexion/extension, abduction/adduction, and axial rotation, which signify movements in sagittal, frontal, and transverse planes, respectively. Under each experimental condition, we extracted the three-dimensional joint angles at the time point of MTC. Then, we calculated the standard deviation of those angles of the ankle, knee, and hip joints in all three planes of the joint coordinate during the 5 minutes of walking. Finally, we performed correlation analyses to investigate any association between the variability of MTC and joint movement.

### Statistical analysis

We used one-way repeated measures analysis of variance (ANOVA) to evaluate significant differences in MTC height, MTC variability, and three-dimensional lower limb joint angles for 17 participants depending on the vibration level (3 levels: No, Sub, and Supra) separately for the dominant and non-dominant leg. We selected Bonferroni correction as the post-hoc test for multiple pairwise comparisons. We tested the assumption of sphericity using Mauchly’s test. If the assumption of sphericity was violated, the Greenhouse-Geisser criterion was used to reduce the degrees of freedom. We performed Pearson’s correlation analysis to assess the strength of the linear relationship between MTC variability and joint movement variability separately for the ankle, knee, and hip joint. The level of statistical significance was set at p<0.05.

## Results

### Minimum toe clearance (MTC) height and variability

**Fig 3:**
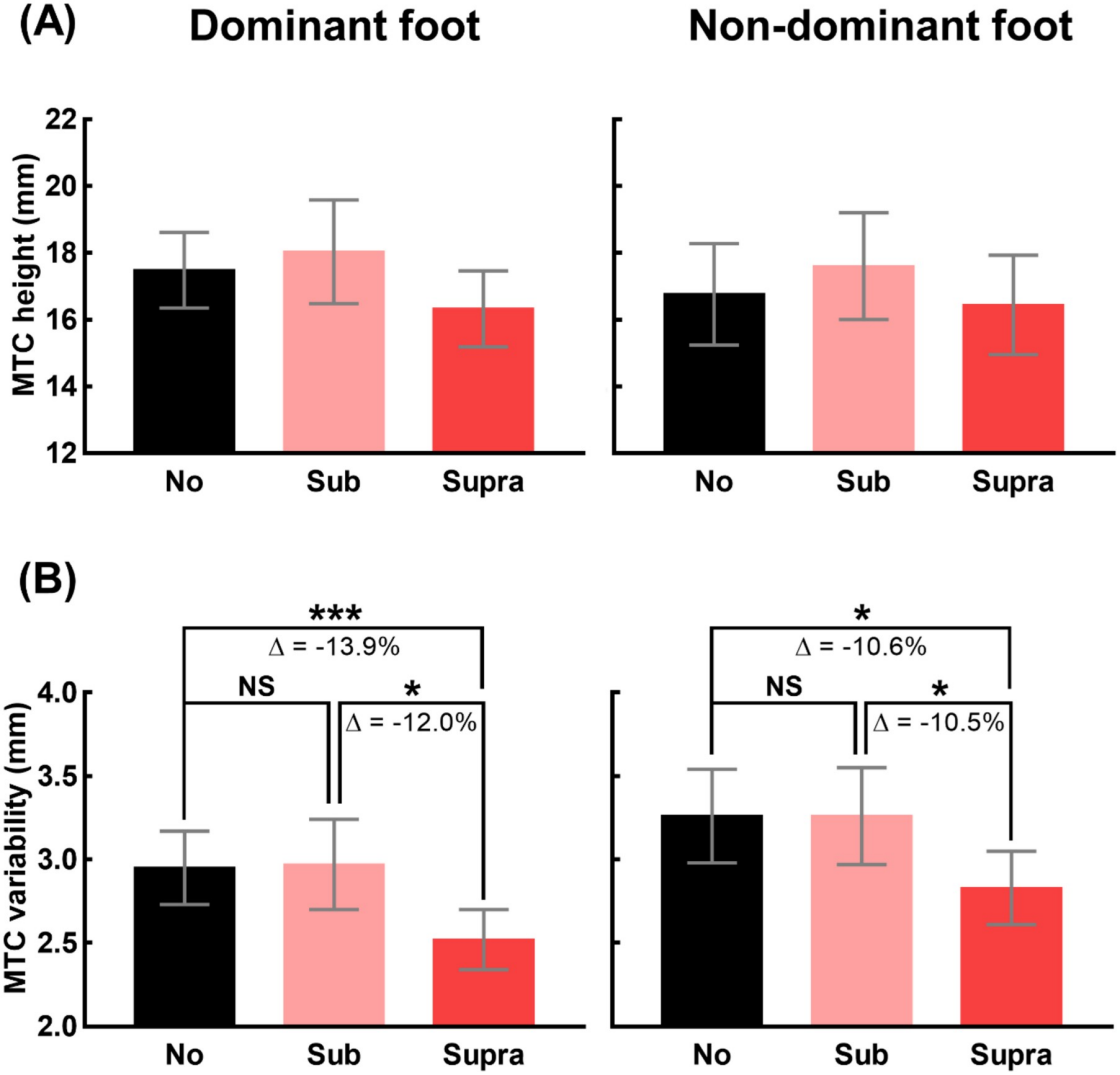
Changes in MTC distribution due to vibrations. (A) and (B) show the means and standard error bars of the values of MTC height and variability of 17 participants, respectively, for the three vibration levels (No: no vibration, Sub: sub-threshold vibration, and Supra: supra-threshold vibration) and both feet. The triple and single asterisk indicate statistically significant difference; ***: p<0.001, and *:p<0.05, whereas NS indicates no statistically significant difference. The reduction in the variability with statistical significance is quantified as Δ (%), which is the ratio of the difference to the larger one.

Fig 3 shows the mean and standard error of the MTC height and its variability of 17 participants under the three vibration levels for both feet. One-way repeated measures ANOVA revealed that there was no significant main effect of vibration level on the values of MTC height for both feet (dominant: F_[1.309, 20.949]_ = 3.875, p = 0.053; non-dominant: F_[1.432, 22.919]_ = 3.131, p = 0.077).

In contrast, a significant main effect of vibration level on the values of MTC variability of 17 participants was observed (dominant: F_[1.485, 23.765]_ = 7.421, p = 0.002; non-dominant: F_[2, 32]_ = 6.048, p = 0.006). For both feet, pairwise comparisons revealed that MTC variability under Supra condition was significantly lower than the variability under No (dominant: p < 0.001, non-dominant: p = 0.026) or Sub (dominant: p = 0.024, non-dominant: p = 0.023) condition. The reduction of the MTC variability (due to the supra-threshold vibration) with respect to the variability under No or Sub condition is indicated as Δ (%) in Fig 3. For the both feet, the average magnitudes of Δ were all greater than 10%.

### Lower limb joint angles and variability of joint angles

**Fig 4:**
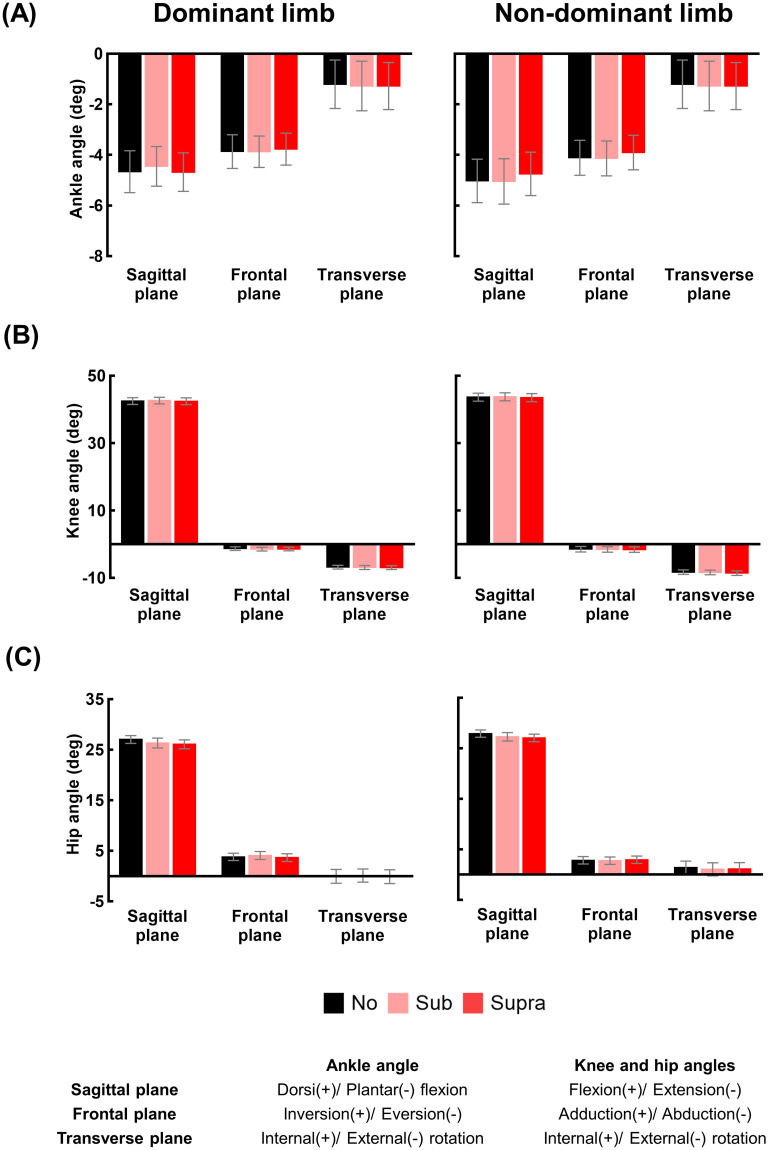
Changes in the lower limb joint angles at MTC due to vibrations. (A-C) show the means and standard errors of the ankle, knee, and hip angles at MTC of 17 participants, respectively, for the three vibration levels (No: no vibration, Sub: sub-threshold vibration, and Supra: supra-threshold vibration) and both feet.

**Fig 5:**
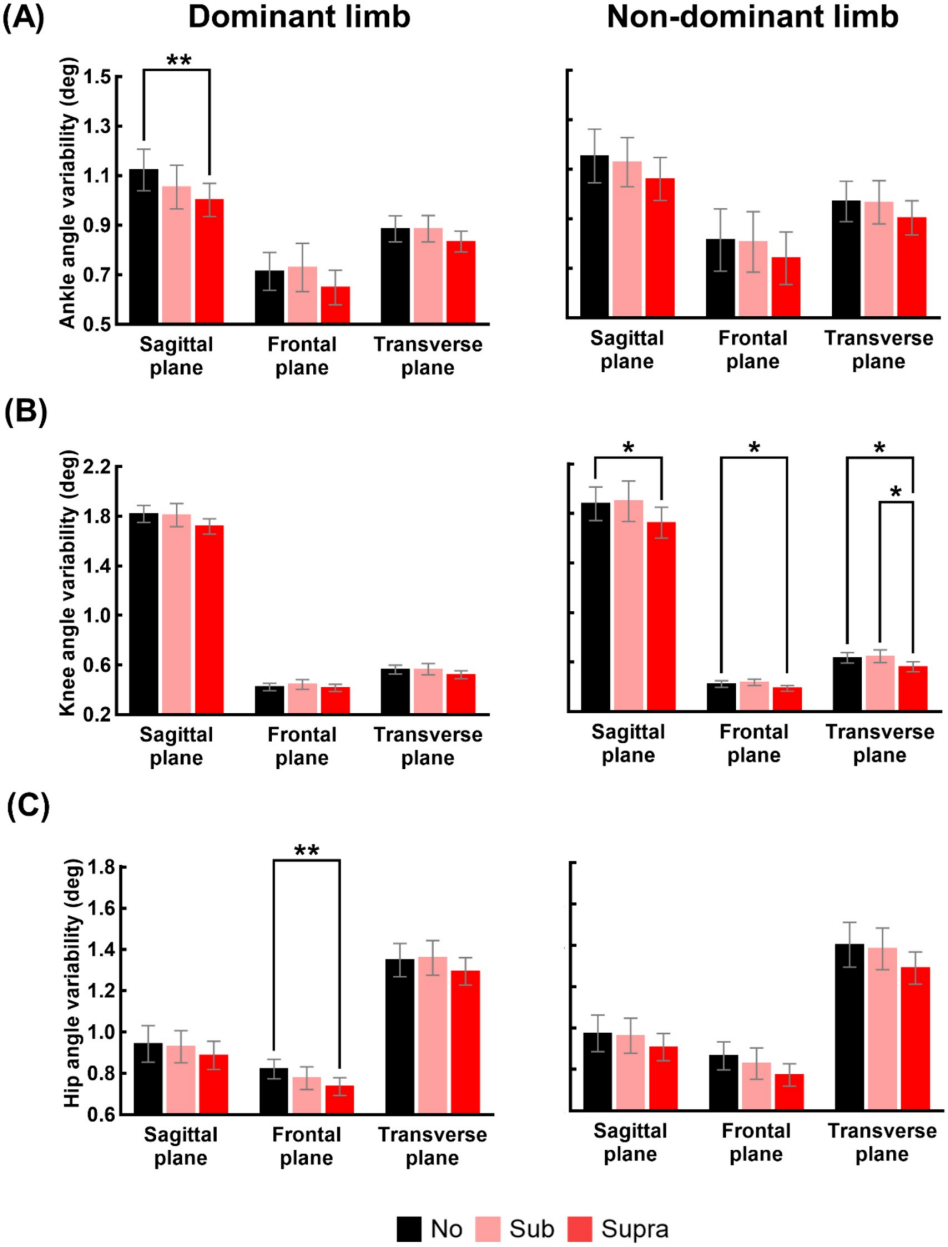
Changes in the variability of lower limb joint angles at MTC due to vibrations. (A-C) show the means and standard errors of the values of the ankle, knee, and hip angles variability at MTC of 17 participants, respectively, for the three vibration levels (No: no vibration, Sub: sub-threshold vibration, and Supra: supra-threshold vibration) and both feet. The double and single asterisk indicate statistically significant difference; **: p<0.01, and *: p<0.05.

Figs 4 and 5 respectively show the mean and standard deviation of the lower limb joint angles and their variability under the three vibration levels. The results of the one-way repeated measures ANOVA evaluating the effect of the vibration level on the lower limb joint angles for 17 participants are compiled in S1 Table of S1 File. Significant main effects of vibration level on the hip angles in the sagittal plane (F_[2, 32]_ = 3.568, p = 0.040) and knee and hip angles in the frontal plane (knee: F_[2, 32]_ = 3.331, p = 0.048; hip: F_[2, 32]_ = 4.623, p = 0.017) were observed for the dominant limb. However, pairwise comparisons revealed no significant difference in the lower limb joint angles between vibration levels.

The results of the one-way repeated measures ANOVA evaluating the effect of the vibration level on the variability of lower limb joint angles for 17 participants are compiled in S2 Table of S1 File. Significant main effects of vibration level on the ankle angle variability in the sagittal plane (F_[2, 32]_ = 6.328, p = 0.005) and knee angle variability in the frontal plane (F_[2, 32]_ = 5.060, p = 0.012) were observed for the dominant limb. For the non-dominant limb, significant main effects of vibration level on the ankle angle variability in the transverse plane (F_[2, 32]_ = 3.935, p = 0.030) and knee angle variability in the all planes (sagittal: F_[2, 32]_ = 4.070, p = 0.027; frontal: F_[2, 32]_ = 6.550, p = 0.004; transverse: F_[2, 32]_ = 8.768, p = 0.001) were observed. Pairwise comparisons revealed that the ankle angle variability in the sagittal plane and hip angle variability in the frontal plane under Supra condition were significantly lower than the variability under No condition for the dominant limb. For non-dominant limb, the knee angle variability values in all three planes under Supra condition were significantly lower than those under No conditions. The knee angle variability in the transverse plane under Supra condition was also significantly lower than that under Sub condition for the non-dominant limb.

### Correlation between MTC and lower limb joint angles variability

We compiled the coefficient of determination (*R*^*2*^) and correlation coefficient (*r*) in Table 1. Significant positive correlations were observed between the variability of MTC and all lower limb joint angles in all three planes, but *R*^*2*^ and *r* for the sagittal planes were higher than those for the frontal and transverse planes. In addition, *R*^*2*^ and *r* for the ankle were higher than those for knee and hip joints. Accordingly, the values of ankle angle variability in the sagittal plane explained the largest proportion of variance in MTC variability and had the strongest linear relationship with MTC variability.

**Table 1:** Pearson’s correlation between minimum toe clearance (MTC) variability and the variability of lower limb joint angles.

Joint	Sagittal plane	Frontal plane	Transverse plane
**Ankle**	*R*^*2*^ = 0.744	*R*^*2*^ = 0.384	*R*^*2*^ = 0.482
	*r* = 0.864***	*r* = 0.624***	*r* = 0.698***
**Knee**	*R*^*2*^ = 0.557	*R*^*2*^ = 0.084	*R*^*2*^ = 0.214
	*r* = 0.749***	*r* = 0.305***	*r* = 0.471***
**Hip**	*R*^*2*^ = 0.428	*R*^*2*^ = 0.375	*R*^*2*^ = 0.340
	*r* = 0.659***	*r* = 0.617***	*r* = 0.588***

*R*^*2*^: coefficient of determination; *r*: correlation coefficient.

*** (p<0.001) denotes a statistically significant correlation between MTC and lower limb joint angles variabilities.

## Discussion

In this study, we demonstrated the efficacy of vibration applied to the soles in reducing MTC variability. The application of supra-threshold vibration significantly decreased MTC variability, whereas the sub-threshold vibration induced no significant change. Further, supra-threshold vibration reduced the variability of three-dimensional joint angles for all three lower body joints. This suggests that the application of vibration to the soles of feet not only reduced MTC variability but also improved movement invariance for all the joints in the lower limb kinematic chain. The main result supports that applying properly designed supra-threshold vibration on the soles using the proposed compact wearable technology can be an effective method for reducing MTC variability during walking.

The vibration-induced increase in sensitivity and/or consistency is a possible explanation for the reduced variability of MTC. Noise added to the sensorimotor system acts as a pedestal to increase the detectability of weak mechanical signals [25, 26, 41]. The applied vibration might induce stochastic resonance (SR) and augment the sensorimotor integration, which in turn decreased MTC variability during walking. A similar reduction in variability in balance and gait parameters was observed when vibration was applied on the end-effectors of the young, elderly, and neurologically impaired individuals [28, 30–32]. Previous studies also suggest vibratory noise can elicit more robustly synchronized activation between the cortical and spinal neurons and enhance motor control. Vibration stimulations on end-effectors and the plausibly resulting SR were reported to significantly improve kinematic and muscular level motor performance consistency for healthy young adults and patients with enhanced physiological tremors [42–44]. This enhanced synchronous activation is consistent with the reduced variability of lower limb joints movements in all three planes (Fig 5).

We analyzed the association between reduction in MTC variability and three-dimensional joint movement variability. Compiling the data for all three vibration conditions and both limbs showed the strongest positive correlation between MTC and sagittal plane ankle joint angle variabilities. The result is predictable considering that the prime movers controlling ankle joint movement in the sagittal plane predominantly mediate MTC [45, 46]. However, interestingly, frontal and transverse plane angular variabilities and the three plane proximal joint angle variabilities also had strong linear correlations with MTC variability. Our results contrast with the results of a previous study by Carter et al., which reported a strong linear association only between MTC variability and sagittal plane hip angle variability for older adults [35]. The clear difference in MTC variability and joint movement patterns between the elderly and healthy young adults may be one source of this contrast.

We deliberately explored the effect of both sub and supra-threshold vibration on the variability of MTC. In our results, only supra-threshold vibration induced significant changes in MTC variability for healthy young adults. Numerous studies reported that optimum noise levels maximizing human motor performance augmentation vary with age and onset of diseases [26, 43, 44]. The selected level of supra-threshold vibration (130%) might be an adequate level of noise that can effectively reduce MTC variability for healthy young adults, whereas the 90% of the threshold might be not. On the other hand, previous studies have reported a decreased variability of spatio-temporal gait parameters for the healthy elderly and recurrent older fallers after the application of sub-sensory vibration [30, 32]. This may be attributed to the enhanced benefit of weaker vibratory noise for degraded somatosensation, which is also prevalent among individuals suffering from stroke, diabetic neuropathy, and Parkinson’s disease [47–49]. In the future, further studies can also address the feasibility of applying sub-sensory vibration to soles to reduce MTC variability for the elderly and people suffering from neurological diseases.

Multiple limitations need to be clarified. First, the information from the current study does not enable us to specify the underlying mechanism of the reduction in the MTC variability. In particular, we did not monitor the spread of vibration beyond the soles of feet. During the swing phase, the lack of pressure and damping between the shoe insole and and foot sole might cause a larger spread of vibration to the proximal segments. The spreaded vibration could also stimulate the sensory organs present at the intrinsic foot and calf muscles, which could possibly affect the MTC variability when supra-threshold vibration was applied. Previous studies have actually shown that non-noxious stimulation applied to the soles of feet can modulate foot and limb trajectory by modifying the muscle reflexes of the lower limb muscles [15, 22, 23] though the vibration delivered in these studies had a single frequency and the amplitude higher than what we used in our study. Additional information from future studies is necessary to systematically quantify or rule out any contribution of various possible mechanisms to the observed vibration-induced reduction in the MTC variability.

Second, we confined the participants to young and healthy adults in this initial study; experiments with the elderly or patients need to be performed in the future work before claiming the efficacy of the proposed intervention for those groups. Third, owing to the limitation of the available infrastructure, we assessed MTC during treadmill walking as in the case of most previous studies. However, there exists difference between the treadmill and over ground walking [50]; a future study may be required to confirm the efficacy of vibration in modifying MTC distribution during over ground walking.

Finally, in this initial study, we selected only two specific levels of vibration (90% and 130% of the sensory threshold), consulting a previous study, which reported these two levels as the most beneficial sub and supra-threshold values enhancing balance during a challenging postural control task [38]. However, considering the difference between walking and balancing, it cannot be guaranteed that these values are also optimal for walking; the 90% of the sensory threshold actually induced no significant effect. This absence of the efficacy of the sub-threshold vibration might imply that the 90% of the sensory threshold “in a sitting position” was not an adequate level of noisy stimulation that could elicit significant effect on “walking.” Multiple previous studies postulated that noise level below an optimal range does not have significant effect on motor performance [44, 51, 52]. Considering that the sensory threshold while standing or walking is higher than the threshold while sitting, the sub-threshold vibration we applied was probably even lower than 90% of the actual threshold during walking, being below the lower boundary that can affect motor behavior. Further elaborated studies for finding the optimal level of vibration stimulation that minimizes MTC variability may augment the efficacy of the proposed intervention further.

The results of this study and the proposed method for reducing MTC variability have important practical implications. The soles of our feet are the body’s closest point of interaction with the ground and contain large number of mechanoreceptors, which provides essential afferent feedback to generate rhythmic gait pattern necessary to regulate balance during walking. In this study, we particularly extracted MTC distribution, a salient indicator of tripping. Our study is the first to show that perceptible vibration to soles can modulate MTC distribution by reducing the variability. It is also noteworthy that the proposed method for delivering the proper stimulation using actuator-embedded shoes is much more practical and compact than the interventions used in previous studies.

## Supporting information

S1 File (DOCX)

S1 Data Manuscript data. The obtained data of the minimum toe clearance (MTC) heights and variabilities, and three-dimension joint angles for ankle, knee, and hip.
(XLSX)

## References

[1] Winter DA. Foot trajectory in human gait: a precise and multifactorial motor control task. Phys Ther. 1992;72(1):45–53. doi: 10.1093/ptj/72.1.45 1728048

[2] Ivanenko YP, Grasso R, Macellari V, Lacquaniti F. Control of foot trajectory in human locomotion: role of ground contact forces in simulated reduced gravity. J Neurophysiol. 2002;87(6):3070–89. doi: 10.1152/jn.2002.87.6.3070 12037209

[3] Begg R, Best R, Dell’Oro L, Taylor S. Minimum foot clearance during walking: strategies for the minimisation of trip-related falls. Gait Posture. 2007;25(2):191–8. doi: 10.1016/j.gaitpost.2006.03.008 16678418

[4] Schulz BW. Minimum toe clearance adaptations to floor surface irregularity and gait speed. J Biomech. 2011;44(7):1277–84. doi: 10.1016/j.jbiomech.2011.02.010 21354576 PMC5375113

[5] Kitagawa N, Ogihara N. Estimation of foot trajectory during human walking by a wearable inertial measurement unit mounted to the foot. Gait Posture. 2016;45:110–4. doi: 10.1016/j.gaitpost.2016.01.014 26979891

[6] Mills PM, Barrett RS, Morrison S. Toe clearance variability during walking in young and elderly men. Gait Posture. 2008;28(1):101–7. doi: 10.1016/j.gaitpost.2007.10.006 18093833

[7] Alcock L, Galna B, Lord S, Rochester L. Characterisation of foot clearance during gait in people with early Parkinson׳ s disease: deficits associated with a dual task. J Biomech. 2016;49(13):2763–9. doi: 10.1016/j.jbiomech.2016.06.007 27363617

[8] Suda EY, Matias AB, Bus SA, Sacco IC. Impact of diabetic neuropathy severity on foot clearance complexity and variability during walking. Gait Posture. 2019;74:194–9. doi: 10.1016/j.gaitpost.2019.09.014 31550557

[9] Tirosh O, Cambell A, Begg RK, Sparrow WA. Biofeedback training effects on minimum toe clearance variability during treadmill walking. Ann Biomed Eng. 2013;41(8):1661–9. doi: 10.1007/s10439-012-0673-6 23064822

[10] Begg RK, Tirosh O, Said CM, Sparrow WA, Steinberg N, Levinger P, et al. Gait training with real-time augmented toe-ground clearance information decreases tripping risk in older adults and a person with chronic stroke. Front Hum Neurosci. 2014;8:243. doi: 10.3389/fnhum.2014.00243 24847234 PMC4021142

[11] Pearson KG. Generating the walking gait: role of sensory feedback. Prog Brain Res. 2004;143:123–9. doi: 10.1016/S0079-6123(03)43012-4 14653157

[12] Horak FB. Postural orientation and equilibrium: what do we need to know about neural control of balance to prevent falls? Age Ageing. 2006;35(suppl_2):ii7–ii11. doi: 10.1093/ageing/afl077 16926210

[13] MacKinnon CD. Sensorimotor anatomy of gait, balance, and falls. Handbook of clinical neurology. 2018;159:3–26. doi: 10.1016/B978-0-444-63916-5.00001-X 30482322 PMC7069605

[14] Inglis JT, Kennedy PM, Wells C, Chua R. The role of cutaneous receptors in the foot. Sensorimotor control of movement and posture: Springer; 2002. p. 111–7. 10.1007/978-1-4615-0713-0_14 12171100

[15] Pearcey GE, Zehr EP. We are upright-walking cats: human limbs as sensory antennae during locomotion. Physiology. 2019;34(5):354–64. doi: 10.1152/physiol.00008.2019 31389772

[16] Nurse MA, Nigg BM. The effect of changes in foot sensation on plantar pressure and muscle activity. Clin Biomech. 2001;16(9):719–27. doi: 10.1016/s0268-0033(01)00090-0 11714548

[17] Eils E, Nolte S, Tewes M, Thorwesten L, Völker K, Rosenbaum D. Modified pressure distribution patterns in walking following reduction of plantar sensation. J Biomech. 2002;35(10):1307–13. doi: 10.1016/s0021-9290(02)00168-9 12231276

[18] Taylor AJ, Menz HB, Keenan A-M. Effects of experimentally induced plantar insensitivity on forces and pressures under the foot during normal walking. Gait Posture. 2004;20(3):232–7. doi: 10.1016/j.gaitpost.2003.02.001 15531169

[19] Yang JF, Stein RB. Phase-dependent reflex reversal in human leg muscles during walking. J Neurophysiol. 1990;63(5):1109–17. doi: 10.1152/jn.1990.63.5.1109 2358865

[20] Van Wezel BM, Ottenhoff FA, Duysens J. Dynamic control of location-specific information in tactile cutaneous reflexes from the foot during human walking. J Neurosci. 1997;17(10):3804–14. doi: 10.1523/JNEUROSCI.17-10-03804.1997 9133399 PMC6573668

[21] Nakajima T, Sakamoto M, Tazoe T, Endoh T, Komiyama T. Location specificity of plantar cutaneous reflexes involving lower limb muscles in humans. Experimental brain research. 2006;175(3):514–25. doi: 10.1007/s00221-006-0568-6 16847613

[22] Duysens J, Trippel M, Horstmann G, Dietz V. Gating and reversal of reflexes in ankle muscles during human walking. Exp Brain Res. 1990;82(2):351–8. doi: 10.1007/BF00231254 2286237

[23] Zehr EP, Nakajima T, Barss T, Klarner T, Miklosovic S, Mezzarane RA, et al. Cutaneous stimulation of discrete regions of the sole during locomotion produces “sensory steering” of the foot. BMC Sports Sci Med Rehabilitation. 2014;6(1):1–21. 10.1186/2052-1847-6-33 PMC4158001 25202452

[24] Collins J, Chow CC, Imhoff TT. Stochastic resonance without tuning. Nature. 1995;376(6537):236–8. doi: 10.1038/376236a0 7617033

[25] Collins JJ, Imhoff TT, Grigg P. Noise-enhanced tactile sensation. Nature. 1996. doi: 10.1038/383770a0 8893000

[26] Harry JD, Niemi JB, Priplata AA, Collins J. Balancing act [noise based sensory enhancement technology]. IEEE Spectr. 2005;42(4):36–41.

[27] Priplata AA, Niemi JB, Harry JD, Lipsitz LA, Collins JJ. Vibrating insoles and balance control in elderly people. Lancet. 2003;362(9390):1123–4. doi: 10.1016/S0140-6736(03)14470-4 14550702

[28] Priplata AA, Patritti BL, Niemi JB, Hughes R, Gravelle DC, Lipsitz LA, et al. Noise-enhanced balance control in patients with diabetes and patients with stroke. Ann Neurol. 2006;59(1):4–12. doi: 10.1002/ana.20670 16287079

[29] Hijmans JM, Geertzen J, Zijlstra W, Hof AL, Postema K. Effects of vibrating insoles on standing balance in diabetic neuropathy. J Rehabil Res Dev. 2008;45:1442–50. 19319766

[30] Lipsitz LA, Lough M, Niemi J, Travison T, Howlett H, Manor B. A shoe insole delivering subsensory vibratory noise improves balance and gait in healthy elderly people. Arch Phys Med Rehabil. 2015;96(3):432–9. doi: 10.1016/j.apmr.2014.10.004 25450133 PMC4339481

[31] Stephen DG, Wilcox BJ, Niemi JB, Franz J, Kerrigan DC, D’Andrea SE. Baseline-dependent effect of noise-enhanced insoles on gait variability in healthy elderly walkers. Gait Posture. 2012;36(3):537–40. doi: 10.1016/j.gaitpost.2012.05.014 22739049 PMC3978195

[32] Galica AM, Kang HG, Priplata AA, D’Andrea SE, Starobinets OV, Sorond FA, et al. Subsensory vibrations to the feet reduce gait variability in elderly fallers. Gait Posture. 2009;30(3):383–7. doi: 10.1016/j.gaitpost.2009.07.005 19632845 PMC2745077

[33] Yamashita S, Igarashi K, Ogihara N. Reducing the foot trajectory variabilities during walking through vibratory stimulation of the plantar surface of the foot. Sci Rep. 2021;11(1):1–8. 33782523 10.1038/s41598-021-86583-7 PMC8007736

[34] Moon J, Pathak P, Kim S, Roh S-g, Roh C, Shim Y, et al. Shoes with active insoles mitigate declines in balance after fatigue. Sci Rep. 2020;10(1):1–11. 32029789 10.1038/s41598-020-58815-9 PMC7004992

[35] Carter SC, Batavia MZ, Gutierrez GM, Capezuti EA. Joint movements associated with minimum toe clearance variability in older adults during level overground walking. Gait Posture. 2020;75:14–21. doi: 10.1016/j.gaitpost.2019.09.025 31586752

[36] Faul F, Erdfelder E, Buchner A, Lang A-G. Statistical power analyses using G* Power 3.1: tests for correlation and regression analyses. Behav Res Methods. 2009;41(4):1149–60. doi: 10.3758/BRM.41.4.1149 19897823

[37] Mildren RL, Strzalkowski ND, Bent LR. Foot sole skin vibration perceptual thresholds are elevated in a standing posture compared to sitting. Gait Posture. 2016;43:87–92. doi: 10.1016/j.gaitpost.2015.10.027 26669957

[38] Severini G, Delahunt E. Effect of noise stimulation below and above sensory threshold on postural sway during a mildly challenging balance task. Gait Posture. 2018;63:27–32. doi: 10.1016/j.gaitpost.2018.04.031 29704801

[39] Meyer C, Killeen T, Easthope CS, Curt A, Bolliger M, Linnebank M, et al. Familiarization with treadmill walking: How much is enough? Sci Rep. 2019;9(1):1–10. 30914746 10.1038/s41598-019-41721-0 PMC6435738

[40] Zeni J Jr, Richards J, Higginson J. Two simple methods for determining gait events during treadmill and overground walking using kinematic data. Gait Posture. 2008;27(4):710–4. doi: 10.1016/j.gaitpost.2007.07.007 17723303 PMC2384115

[41] Lirani-Silva E, Beretta VS, Jimenez AMF, Gobbi LTB. Postural control and somatosensory information: effects of aging and Parkinson’s disease. Locomotion and Posture in Older Adults: Springer; 2017. p. 307–22.

[42] Trenado C, Amtage F, Huethe F, Schulte-Mönting J, Mendez-Balbuena I, Baker SN, et al. Suppression of enhanced physiological tremor via stochastic noise: initial observations. PLoS One. 2014;9(11):e112782. doi: 10.1371/journal.pone.0112782 25397577 PMC4232445

[43] Trenado C, Mikulić A, Manjarrez E, Mendez-Balbuena I, Schulte-Mönting J, Huethe F, et al. Broad-band Gaussian noise is most effective in improving motor performance and is most pleasant. Front Hum Neurosci. 2014;8:22. doi: 10.3389/fnhum.2014.00022 24550806 PMC3910318

[44] Mendez-Balbuena I, Manjarrez E, Schulte-Mönting J, Huethe F, Tapia JA, Hepp-Reymond M-C, et al. Improved sensorimotor performance via stochastic resonance. J Neurosci. 2012;32(36):12612–8. doi: 10.1523/JNEUROSCI.0680-12.2012 22956850 PMC6621271

[45] De Asha AR, Buckley JG. The effects of walking speed on minimum toe clearance and on the temporal relationship between minimum clearance and peak swing-foot velocity in unilateral trans-tibial amputees. Prosthet Orthot Int. 2015;39(2):120–5. doi: 10.1177/0309364613515493 24469428 PMC4361493

[46] Perera CK, Ahmad SA, Gouwanda D. Muscles affecting minimum toe clearance. Front Public Health. 2021;9:668. doi: 10.3389/fpubh.2021.612064 34136448 PMC8200481

[47] Menz HB, Lord SR, St George R, Fitzpatrick RC. Walking stability and sensorimotor function in older people with diabetic peripheral neuropathy. Arch Phys Med Rehabil. 2004;85(2):245–52. doi: 10.1016/j.apmr.2003.06.015 14966709

[48] Wing K, Lynskey JV, Bosch PR. Walking speed in stroke survivors: considerations for clinical practice. Top Geriatr Rehabil. 2012;28(2):113–21.

[49] Nelson AJ, Premji A, Rai N, Hoque T, Tommerdahl M, Chen R. Dopamine alters tactile perception in Parkinson’s disease. Can J Neurol Sci. 2012;39(1):52–7. doi: 10.1017/s0317167100012683 22384496

[50] Riley PO, Paolini G, Della Croce U, Paylo KW, Kerrigan DC. A kinematic and kinetic comparison of overground and treadmill walking in healthy subjects. Gait Posture. 2007;26(1):17–24. doi: 10.1016/j.gaitpost.2006.07.003 16905322

[51] Treviño M, la Torre-Valdovinos D, Manjarrez E. Noise improves visual motion discrimination via a stochastic resonance-like phenomenon. Front Hum Neurosci. 2016;10:572. doi: 10.3389/fnhum.2016.00572 27932960 PMC5120109

[52] Ribot-Ciscar E, Hospod V, Aimonetti J-M. Noise-enhanced kinaesthesia: a psychophysical and microneurographic study. Exp Brain Res. 2013;228(4):503–11. doi: 10.1007/s00221-013-3581-6 23712687

