# Supplementary material for: Application of vibration to the soles reduces minimum toe clearance variability during walking
Source: PLoS One. 2022 Jan 4;17(1):e0261732. doi: 10.1371/journal.pone.0261732 (PMC8726470; doi:10.1371/journal.pone.0261732)
Supplement: S1 File — (DOCX) [file pone.0261732.s001.docx]

**Supplementary Information**

**S1 Table. The results of one-way repeated measures ANOVA: effects of three vibration level conditions on mean lower limb joint angles at the time point of MTC.**

| **Joint** | | **Within-subject effects** | | |
| --- | --- | --- | --- | --- |
|  |  | **Sagittal plane** | **Frontal plane** | **Transverse plane** |
| **Ankle** | **Dominant** | F_[2,32]_ = 1.050,  p = 0.362 | F_[2,32]_ = 0.978,  p = 0.387 | F_[2,32]_ = 0.232,  p = 0.794 |
|  | **Non-dominant** | F_[2,32]_ = 2.610,  p = 0.089 | F_[2,32]_ = 2.576,  p = 0.092 | F_[2,32]_ = 2.177,  p = 0.130 |
| **Knee** | **Dominant** | F_[2,32]_ = 0.554,  p = 0.580 | **F_[2,32]_ = 3.331,**  **p = 0.048** | F_[2,32]_ = 1.811,  p = 0.180 |
|  | **Non-dominant** | F_[2,32]_ = 1.090,  p = 0.348 | F_[2,32]_ = 1.879,  p = 0.169 | F_[2,32]_ = 4.282,  p = 0.022 |
| **Hip** | **Dominant** | **F_[2,32]_ = 3.568,**  **p = 0.040** | **F_[2,32]_ = 4.623,**  **p = 0.017** | F_[2,32]_ = 0.640,  p = 0.534 |
|  | **Non-dominant** | F_[2,32]_ = 2.517,  p = 0.097 | F_[2,32]_ = 0.800,  p = 0.458 | F_[2,32]_ = 1.504,  p = 0.238 |

| **Joint** | | **Within-subject effects** | | |
| --- | --- | --- | --- | --- |
|  |  | **Sagittal plane** | **Frontal plane** | **Transverse plane** |
| **Ankle** | **Dominant** | **F_[2,32]_ = 6.328,**  **p = 0.005** | F_[2,32]_ = 2.636,  p = 0.087 | F_[2,32]_ = 2.658,  p = 0.086 |
|  | **Non-dominant** | F_[2,32]_ = 2.263,  p = 0.120 | F_[1.419,22.703]_ = 2.298,  p = 0.136 | **F_[2,32]_ = 3.935,**  **p = 0.030** |
| **Knee** | **Dominant** | F_[2,32]_ = 2.440,  p = 0.103 | F_[2,32]_ = 1.149,  p = 0.330 | F_[2,32]_ = 2.363,  p = 0.110 |
|  | **Non-dominant** | **F_[2,32]_ = 4.070,**  **p = 0.027** | **F_[2,32]_ = 6.550,**  **p = 0.004** | **F_[2,32]_ = 8.768,**  **p = 0.001** |
| **Hip** | **Dominant** | F_[2,32]_ = 1.878,  p = 0.169 | **F_[2,32]_ = 5.060,**  **p = 0.012** | F_[2,32]_ = 0.720,  p = 0.494 |
|  | **Non-dominant** | F_[2,32]_ = 1.122,  p = 0.338 | F_[2,32]_ = 3.127,  p = 0.057 | F_[2,32]_ = 2.377,  p = 0.109 |

**S2 Table. The results of one-way repeated measures ANOVA: effects of three vibration level conditions on the variability of lower limb joint angles at the time point of MTC.**

**S1 Methods. Evaluation of the effect of time and three vibration level conditions over every 30s time intervals on the MTC distribution, and the effect of foot dominance on MTC distribution.**

We calculated the average and standard deviation of MTC heights over every thirty-seconds time intervals for the 5 minutes of walking data to assess the changes in MTC height and variability over the trial. These values were calculated separately for each foot and the three vibration level conditions (NO: no vibration, Sub: sub-threshold vibration, and Supra: supra-threshold vibration). We then used two-way repeated measures analysis of variance (ANOVA) to evaluate significant differences in the MTC height and variability for 17 participants depending on time (10 levels: values over every thirty-seconds time interval; 0~30, 30~60, 60~90, 90~120, 120~150, 150~180, 180~210, 210~240, 240~270, and 270~300) and the vibration level (3 levels: No, Sub, and Supra) separately for the dominant and non-dominant foot. We selected Bonferroni correction as the post-hoc test for multiple pairwise comparisons. We tested the assumption of sphericity using Mauchly’s test. If the assumption of sphericity was violated, the Greenhouse-Geisser criterion was used to reduce the degrees of freedom.

To evaluate differences in MTC distribution between feet, we performed paired t-test between the MTC height and variability of dominant and non-dominant foot for 17 participants. The level of statistical significance was set at p < 0.05.

**S1 Results.** **Evaluation of the effect of time and three vibration level conditions over 30s time intervals on the MTC distribution, and the effect of foot dominance on MTC distribution.**

Supplementary Figure S1 shows the mean and standard error of the MTC height of 17 participants under the three vibration level conditions over every 30s time intervals for both feet. Two-way repeated measures ANOVA revealed a significant main effect of time on the values of MTC height only for the non-dominant foot (dominant: F_[3.100, 49.605]_ = 1.893, p = 0.141; non-dominant: F_[3.918, 62.682]_ = 3.593, p = 0.011) whereas, no significant main effect of vibration level was revealed for both feet (dominant: F_[1.298, 20.770]_ = 3.845, p = 0.054; non-dominant: F_[1.431, 22.889]_ = 3.202, p = 0.074). Also, no significant interaction effect was revealed between time and vibration level on the values of MTC height for both feet (dominant: F_[18, 288]_ = 0.962, p = 0.504; non-dominant: F_[18, 288]_ = 0.742, p = 0.767). Pairwise comparisons revealed no significant differences between the values of MTC height at any of the 30s time intervals for the non-dominant foot.

Supplementary Figure S2 shows the mean and standard error of the MTC variability of 17 participants under the three vibration level conditions over every 30s time interval for both feet. Two-way repeated measures ANOVA revealed a significant main effect of time on the values of MTC variability only for the non-dominant foot (dominant: F_[4.385, 70.166]_ = 1.108, p = 0.362; non-dominant: F_[4.172, 66.745]_ = 4.238, p = 0.004), whereas significant main effect of vibration level was revealed for both feet (dominant: F_[2, 32]_ = 12.127, p < 0.001; non-dominant: F_[2, 32]_ = 6.264, p = 0.005). However, no significant interaction effect was revealed between time and vibration level on the values of MTC variability for both feet (dominant: F_[18, 288]_ = 0.925, p = 0.548; non-dominant: F_[18, 288]_ = 1.232, p = 0.235). Pairwise comparisons revealed no significant differences between the values of MTC variability at any of the 30s time intervals for the non-dominant foot. On the other hand, for the dominant foot, pairwise comparisons revealed that MTC variability under Supra condition was significantly lower than the variability under No condition for the 0~30s (p = 0.015), 60~90s (p = 0.007), and 150~180s (p = 0.012) time intervals. On the other hand, for the 30s time intervals between 210 and 300 seconds, MTC variability under Supra condition was significantly lower than the variability under No (210~240: p = 0.048; 240~270: p = 0.001; 270~300: p = 0.023) and Sub (210~240: p = 0.005; 240~270: p = 0.046; 270~300: p = 0.014) conditions. For the non-dominant foot, pairwise comparisons revealed that MTC variability for the 30~60s time intervals under Supra condition was significantly lower than the variability under No (p = 0.021) and Sub (p = 0.049) conditions.


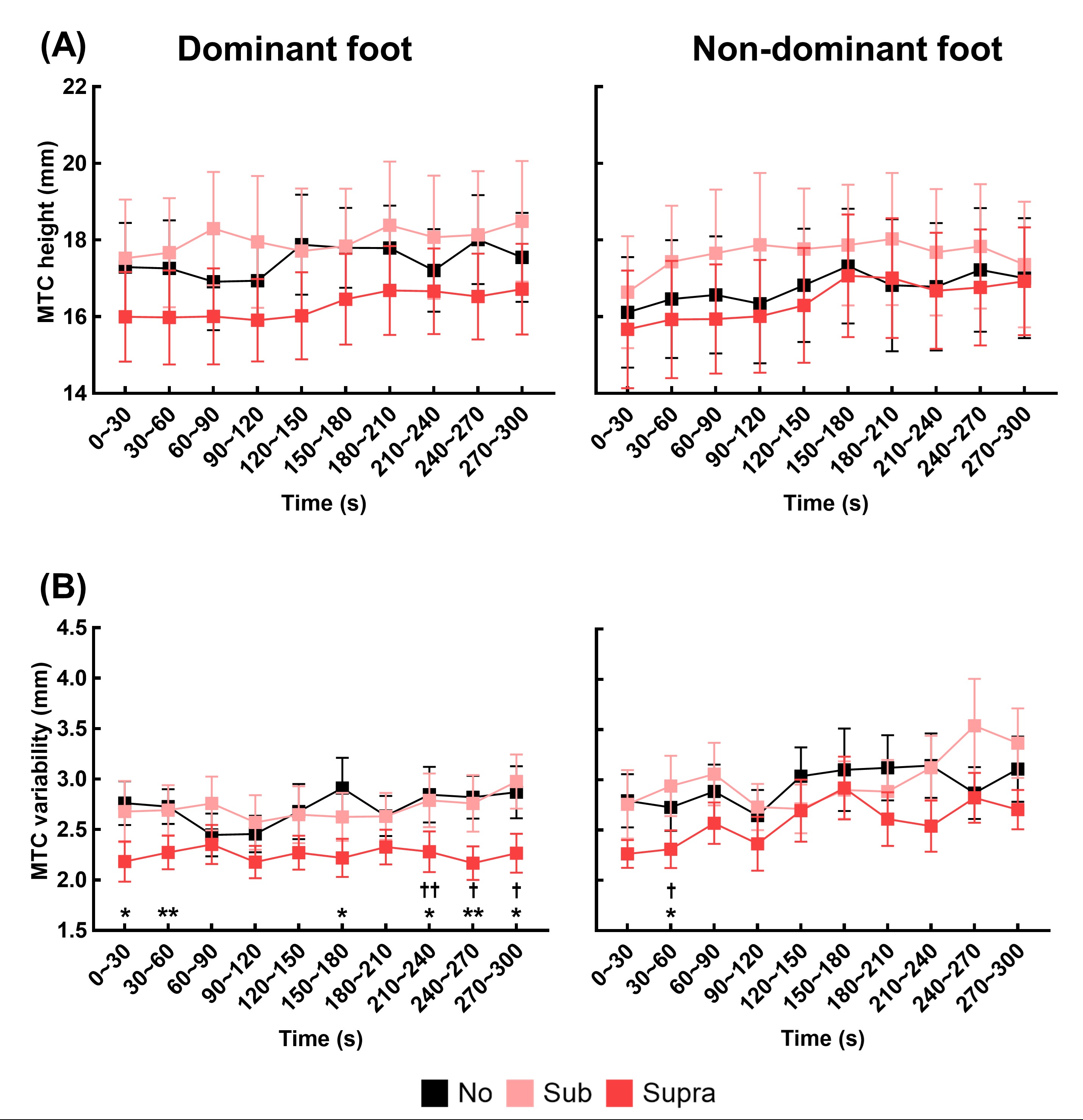


**S1 Fig. Changes in the MTC distribution over 30s time intervals for the 5 minutes walking data.** (A) and (B) show the means and standard error bards of the MTC height and variability of 17 participants, respectively, for the ten 30s time intervals (0~30, 30~60, 60~90, 90~120, 120~150, 150~180, 180~210, 210~240, 240~270, and 270~300s), three vibration levels (No: no vibration, Sub: sub-threshold vibration, and Supra: supra-threshold vibration) and both feet. The asterisk indicates statistically significant differences between No and Supra conditions; **: p<0.01, and *: p<0.05. The cross indicates statistically significant difference between Sub and Supra conditions; ††: p<0.01, and †: p<0.05.


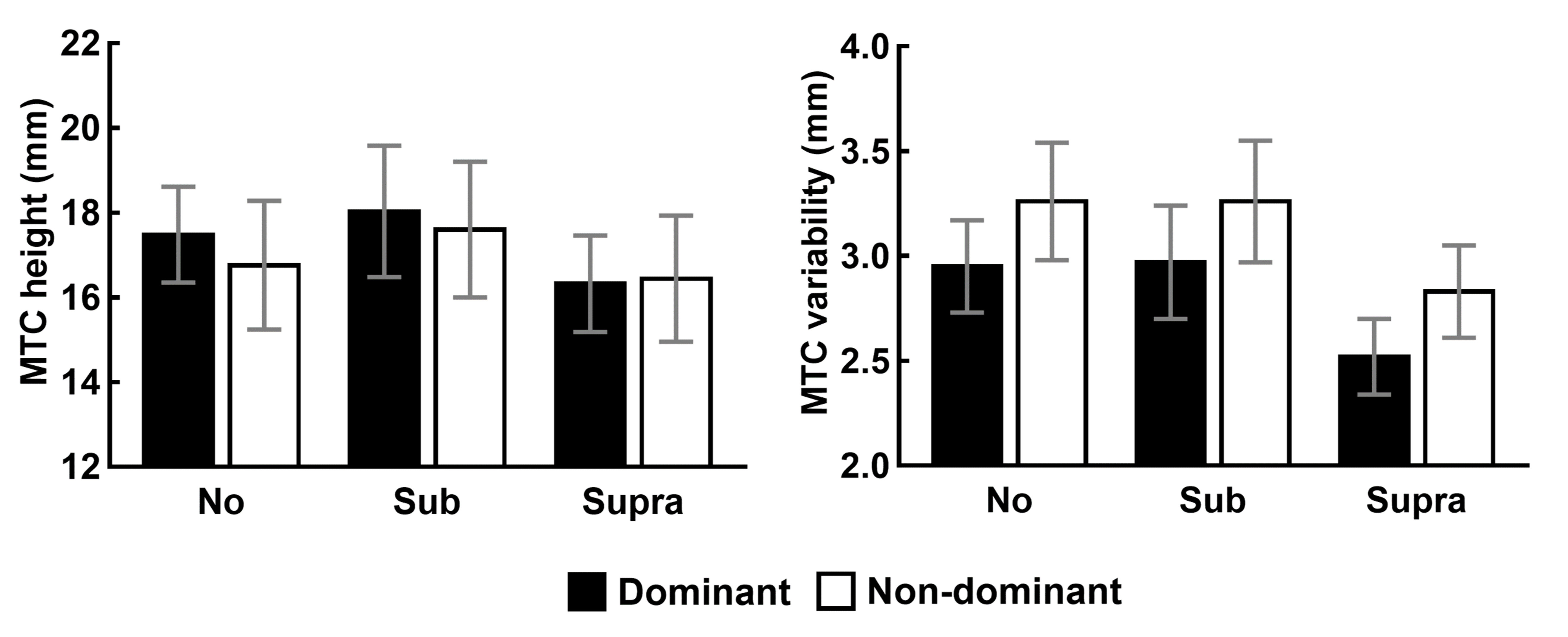


**S2 Fig. Differences in MTC distribution according to foot dominance.** The figure shows the means and standard error bars of the MTC height and variability of 17 participants, respectively, for the three vibration levels (No: no vibration, Sub: sub-threshold vibration, and Supra: supra-threshold vibration) and both feet. Paired t-tests revealed no differences in MTC height and variability between dominant and non-dominant foot for any of the three vibration levels.
